# The Relationship Between Head Motion Synchronization and Empathy in Unidirectional Face-to-Face Communication

**DOI:** 10.3389/fpsyg.2018.01622

**Published:** 2018-09-25

**Authors:** Takahiro Yokozuka, Eisuke Ono, Yuki Inoue, Ken-Ichiro Ogawa, Yoshihiro Miyake

**Affiliations:** ^1^Laboratory of Miyake, Department of Computational Intelligence and Systems Science, Tokyo Institute of Technology, Tokyo, Japan; ^2^Department of Computer Science, Tokyo Institute of Technology, Tokyo, Japan

**Keywords:** non-verbal behavior, multimodal human interaction, body motion synchronization, head motion, empathy

## Abstract

Embodied synchronization is widely observed in human communication, and is considered to be important in generating empathy during face-to-face communication. However, the quantitative relationship between body motion synchronization and degree of empathy is not fully understood. Therefore, we focused on head motion to investigate phase and frequency differences in head motion synchronization in relation to degree of empathy. We specifically conducted a lecture-based experiment using controlled spoken text divided into two parts: high empathy and low empathy. During the lecture, we measured the acceleration of speakers’ and listeners’ head motions using an accelerometer, and calculated the synchronization between the time-series data from their acceleration norms. The results showed greater head motion synchronization during high empathy. During high empathy, the speakers’ head motions began before those of listeners’ in the medium (2.5 to 3.5 Hz) and high (4.0 to 5.0 Hz) frequency ranges, whereas the speakers’ head motions tended to start later than those of the listeners’ in the low (1.0 to 2.0 Hz) and medium (2.5 to 3.5 Hz) frequency ranges. This suggests that the degree of empathy is reflected by a different relationship between the phase and frequency of head motion synchronization during face-to-face communication.

## Introduction

Non-verbal communication channels play an important role for sharing emotional information during human communication. For instance, synchronization of non-verbal behaviors occurs in various forms of social communication, such as between a mother and infant (Meltzoff and Moore, 1983; Bernieri et al., 1988), physician and patient (Koss and Rosenthal, 1997), teacher and student (Bernieri, 1988; Lafrance and Broadbent, 1988), and psychological counselor and client (Ramseyer and Tschacher, 2006; Koole and Tschacher, 2016). In addition, synchronization of non-verbal behaviors has psychologically positive effects (Tickle-Degnen and Rosenthal, 1990; Hall et al., 2012). For instance, body synchronization between a counselor and a client represents their mutual empathy and relates to their level of satisfaction with counseling (Komori and Nagaoka, 2008; Ramseyer and Tschacher, 2014). In a group activity, when a group of students is rhythmically synchronized, they feel rapport with their groupmates, a sense of belonging to the group, and a strong sense of unity (Lakens and Stel, 2011). Body movement imitation between a teacher and students in educational settings leads to higher levels of rapport and greater satisfaction with learning outcomes (Duffy and Chartrand, 2015). Bavelas et al. (1986) and Koehne et al. (2016) reported that greater levels of synchronization are linked to empathy. In addition to the social context, neuroscientific bases have been identified for non-verbal behavior synchronization, such as synchronization of brain activities among participants during successful communication (Stephens et al., 2010) and correlations between body movement and brain activity (Yun et al., 2012).

However, the quantitative relationship between body motion synchronization and degree of empathy is not fully understood. Therefore, to investigate this relationship in greater detail, we analyzed changes in body motion synchronization in relation to the degree of empathy during face-to-face communication, given that it is believed that body motion changes unconsciously with emotional states (Lakin, 2006; de Waal, 2007; Niedenthal, 2007; Richmond et al., 2008).

Clarifying the relationship between changes in physical indicators and degree of empathy would improve interpretation of the cognitive relationship between body motion synchronization and degree of empathy from a physical aspect. Because body motions give different impressions, depending on speed and generation timing (Mehrabian and Williams, 1969; Miller et al., 1976), we specifically analyzed body motion using a set of physical indicators, including frequency and phase difference. We hypothesized that the phase and frequency relationships of body motion synchronization would change according to the degree of empathy during face-to-face communication.

Here, we focused on measuring participants’ head motion acceleration changes while in a seated position. An experiment with a unidirectional (i.e., speaker to listener) face-to-face lecture task was conducted. To manipulate the listeners’ state of empathy during the story, the lecture material was divided into two parts: “low empathy” and “high empathy.” The high empathy part was intended to facilitate listeners’ empathy with the story, in contrast to the low empathy part. After the lecture task, listeners evaluated their degree of empathy with each part of the story. We then statistically compared the incidence of head motion synchronization between speakers and listeners during the low and high empathy conditions. To detect and analyze the head motion synchronization phase and frequency differences, time-series data for each participant’s head motion acceleration were collected using an accelerometer and short-time Fourier analysis; correlation analysis was then used to examine the time-series acceleration norms.

## Materials and Methods

### Ethics Statement

Our experimental protocol was approved by the Ethics Committee of the Tokyo Institute of Technology, and participants were recruited from the Tokyo Institute of Technology. All participants were briefed about the experimental procedures and gave written informed consent prior to participation. The methods were carried out in accordance with the approved guidelines. Informed consent was obtained for publication of identifying images.

### Participants

Forty-eight Japanese adults (22 males, 26 females) were recruited via public advertisements and grouped into 24 same-sex pairs (11 male pairs, 13 female pairs). In each pair, one participant was assigned as the speaker and the other as listener. The participants in each pair had an age difference of no more than 5 years and did not know one another. After the experiment, we checked whether any participant had previously known the content of the material, and excluded one female pair on this basis; none of the other participants had ever read the material before. Interactions between participants before the experiment were not allowed to avoid a familiarity effect between participants.

### Lecture Material

The lecture material, entitled “The meaning of life that our predecessors considered academically,” was adapted from Wikipedia’s (2013) Japanese article “The meaning of life.” The material was divided into two parts, low and high empathy. The low empathy portion related to philosophers’ opinions about the meaning of life, including conceptual and complex sentences and words coined by the philosophers. In contrast, the high empathy portion related to psychologists’ and sociologists’ opinions about the meaning of life using concrete, simple sentences; authors’ names and jargon were deleted from the high empathy sections to make the material easier to understand.

The lecture transcript is included in **Appendix 1**. Each of the two parts included Japanese characters totaling about 650 words. We prepared two versions of the lecture material to eliminate an order effect. Version 1 had low empathy followed by high empathy; version 2 was the reverse order (**Appendix 1** is version 2). For both versions, an identical introduction before the lecture material and conclusion at the end were added. Speaker pairs were randomized to receive version 1 or version 2.

**FIGURE 1:**
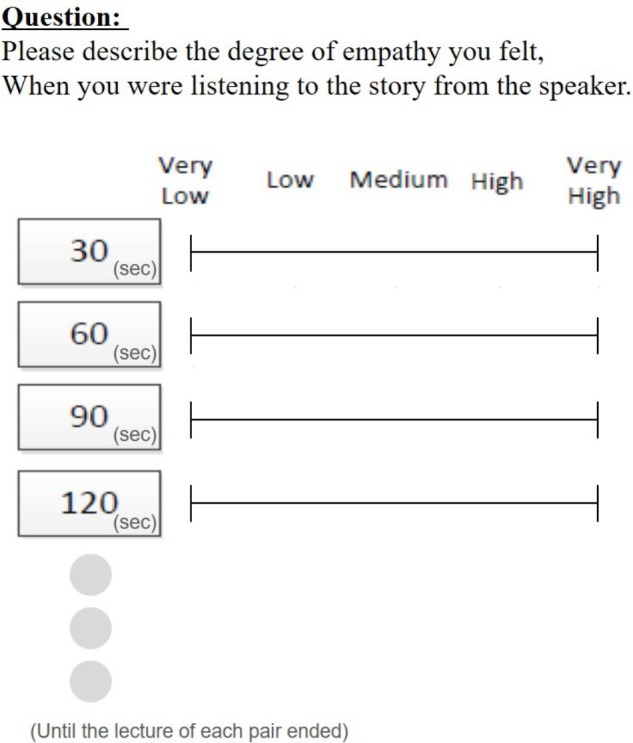
Questionnaire about the listener’s degree of empathy. The listener stopped the video of the lecture every 30 s and filled in their degree of empathy on the questionnaire.

After the experiment, listeners retrospectively watched a video of the lecture and, at 30-s intervals, rated their degree of empathy on a questionnaire using a visual analog scale (**Figure 1**). The results indicate that listeners felt more empathy during the high empathy condition compared with the low empathy condition [*t*(22) = 6.55, ^∗^*p* < 0.01].

### Experiment Environment

**FIGURE 2:**
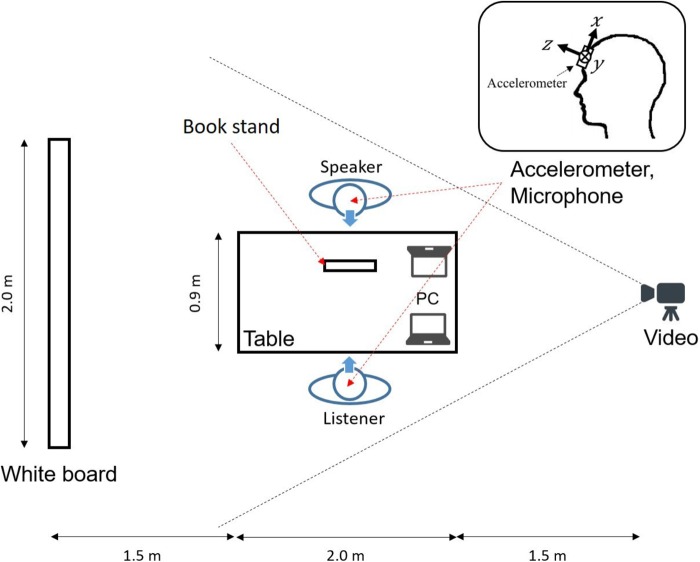
Experiment environment.

**Figure 2** shows the experiment room setup. The speaker and the listener sat in chairs on either side of a table, 0.9 m apart. Environmental factors such as brightness, noise, temperature, and humidity were set at levels suitable for the experiment. A book stand was placed on the table so that the speaker could easily read the lecture material. Wireless accelerometers (WAA-006, Wireless Technology, Inc., Tokyo, Japan; sampling rate: 100 Hz) were attached to the speaker’s and listener’s foreheads with a rubber band to measure time-series data for their head motion accelerations. Since these devices are sufficiently small and light, they did not interfere with participants’ natural movements. The positioning of the accelerometer was selected based on the kinematic perspective that, while in a sitting position, the head moves more frequently compared with other body parts.

### Experiment Procedure

**FIGURE 3:**
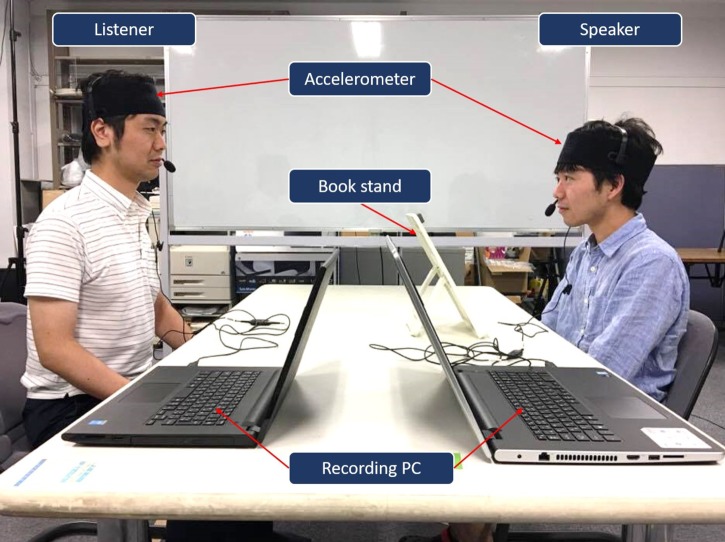
Experimental setting. Written informed consent was obtained from each individual for the publication of this image.

We conducted a lecture experiment using unidirectional face-to-face communication. During the experiment, speakers conveyed lecture material to listeners (**Figure 3**). The speaker was instructed to read the lecture material in a natural way and this took about 5 min. During the experiment, time-series data on head motion acceleration of both the speaker and the listener were measured by their accelerometers.

The experimental procedures were as follows. (1) An investigator explained the experiment to each participant pair in an experiment room. One person in each pair was randomly assigned to the speaker role, and the other the listener role. (2) The listener remained in the experiment room, while the speaker rehearsed the lecture material in a natural way for about 5 min in a separate room. The lecture material was set on a book stand. After the speaker had rehearsed the material, the investigator sat at a table opposite the speaker, and checked whether their reading was clearly audible. (3) The speaker returned to the experiment room. Both the speaker and the listener had an accelerometer and microphone positioned. (4) The investigator cued the participants to begin the experiment and then left the experiment room. (5) During the experiment, the speaker read aloud to the listener, referring to the material set on the book stand, as needed. (6) After the experiment, the speaker and the listener removed their devices.

### Analysis of Head Motion Synchronization

We analyzed the time-series data on head motion acceleration of each participant to detect synchronization using the method described by Thepsoonthorn et al. (2016). This method consists of three main steps.

Step 1: Short-time Frequency Analysis of Acceleration Norms of Head Motion

**FIGURE 4:**
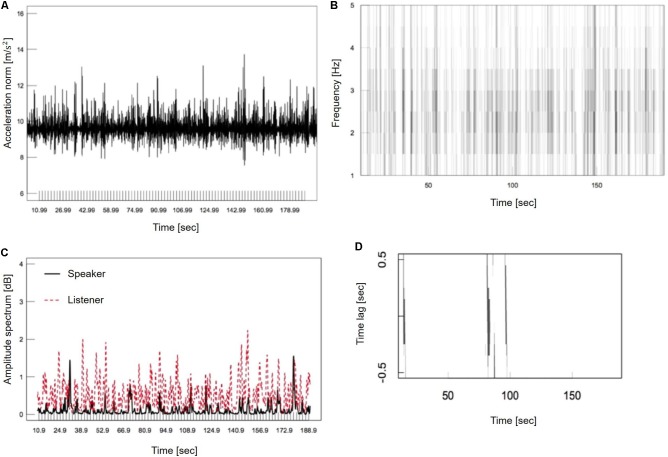
Method of head motion synchronization detection. **(A)** Calculation of head motion acceleration norm. **(B)** Time-frequency analysis of the acceleration norm. **(C)** Amplitude spectrum of 3.0 Hz. **(D)** Identification of head motion synchronization.

First, the normative |a(t)| triaxial acceleration ($a_{x}^{2}$(t),$a_{y}^{2}$(t),$a_{z}^{2}$(t)) of each participant was calculated as:

(1) $$|a(t)|=\sqrt{a_{x}^{2}(t)+a_{y}^{2}(t)+a_{z}^{2}(t)}$$

where the time resolution was set to 0.01 s. **Figure 4A** shows an example of time-series data on the acceleration norm of a participant. Next, a short-time Fourier transform (STFT) was applied to time-series acceleration data as:

(2) $$F(v,t)=\int_{-\infty }^{\infty }a(t)\omega (t^{\prime }-t)\exp(-2\text{π}ivt^{\prime })dt^{\prime }$$

where *v* is frequency in Hz, ω(t) is a Humming window function, and *t* is a central time of window function. In this study, the window width was set to 1.28 s and the window moving time was set to 0.1 s to perform the STFT. Next, the linear interpolation was conducted with respect to frequency *v*. **Figure 4B** shows an example of the STFT within a frequency band of 1.0–5.0 Hz. The darker shade represents a higher amplitude spectrum value. In this study, for detection of head nodding synchronization, the amplitude spectrum was extracted for every 0.5 Hz; that is: 1.0, 1.5, 2.0, 2.5, 3.0, 3.5, 4.0, 4.5, and 5.0 Hz. **Figure 4C** shows the 3.0 Hz amplitude spectrum as an example.

Step 2: Detection of Head Motion Synchronization

The head motion phase difference between speaker and listener was calculated using Spearman’s rank correlation to detect head nodding synchronization. Synchronization between two participants during a tapping task is only possible for the temporal interval of tapping sounds within a range of 200–1800 ms (Fraisse, 1982). Therefore, in this study, the window width was set to 1.8 s, and the frame shift of the window was set to 0.1 s. In addition, the phase difference within a range of −0.5 to +0.5 s was used with a temporal interval of 0.1 s. This was based on a report that in a positive psychotherapeutic session between a client and therapist, the therapist’s body motions occur on a 0.5 s delay (Komori and Nagaoka, 2011). Furthermore, it has been reported that synchronization between infant movements and adult speech occur at a phase difference of 0.05 ± 0.2 s (Kato et al., 1983). There were two criteria for detection of head motion synchronization. First, the amplitude spectrum of each participant’s head motion had a value of more than 90% in the amplitude spectrum throughout the experiment for each pair. Second, for the amplitude spectrum to satisfy the first condition, Spearman’s rank correlation must be positive and statistically significant. **Figure 4D** shows an example of head motion synchronization, where synchronization is represented by the black vertical lines.

Step 3: Calculation of Mean Value and Test of Incidence of Head Motion Synchronization

**FIGURE 5:**
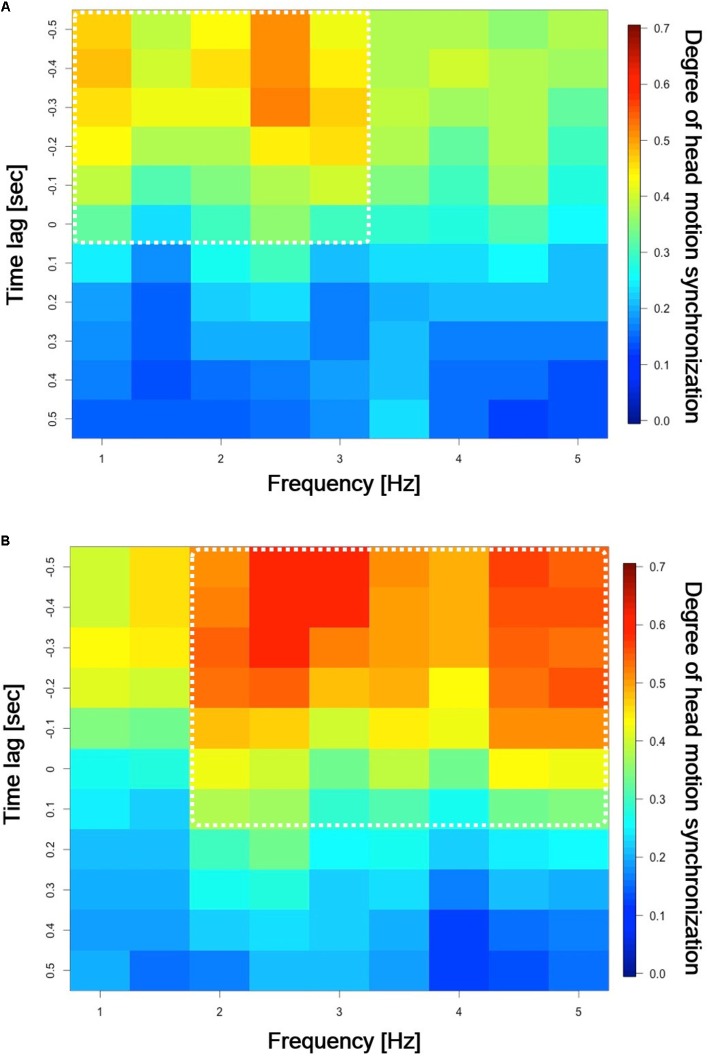
Incidence of head motion synchronization for low and high empathy conditions. The incidence of head motion synchronization is illustrated by a continuous spectrum of colors from red to blue. **(A)** Mean incidence of head motion synchronization during low empathy (*n* = 23). **(B)** Mean incidence of head motion synchronization during high empathy (*n* = 23).

**FIGURE 6:**
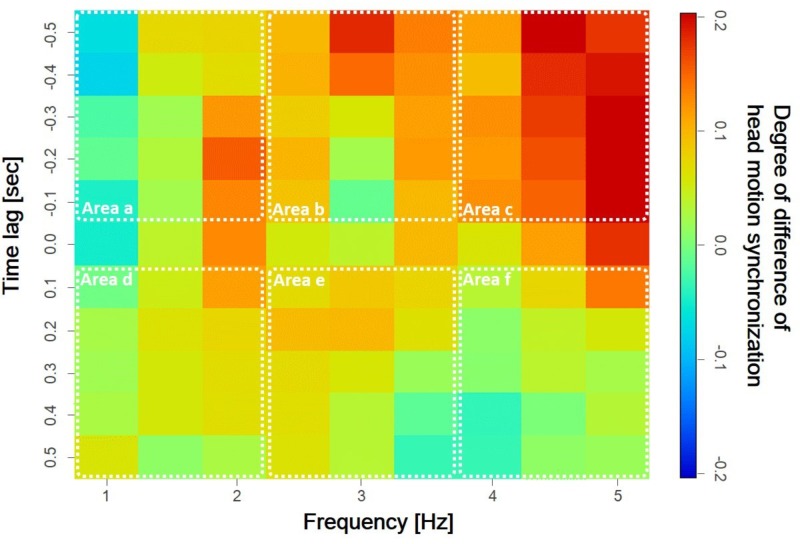
Difference in incidence of head motion synchronization between low and high empathy conditions. The dashed line shows the six areas of comparison between high and low empathy conditions.

**FIGURE 7:**
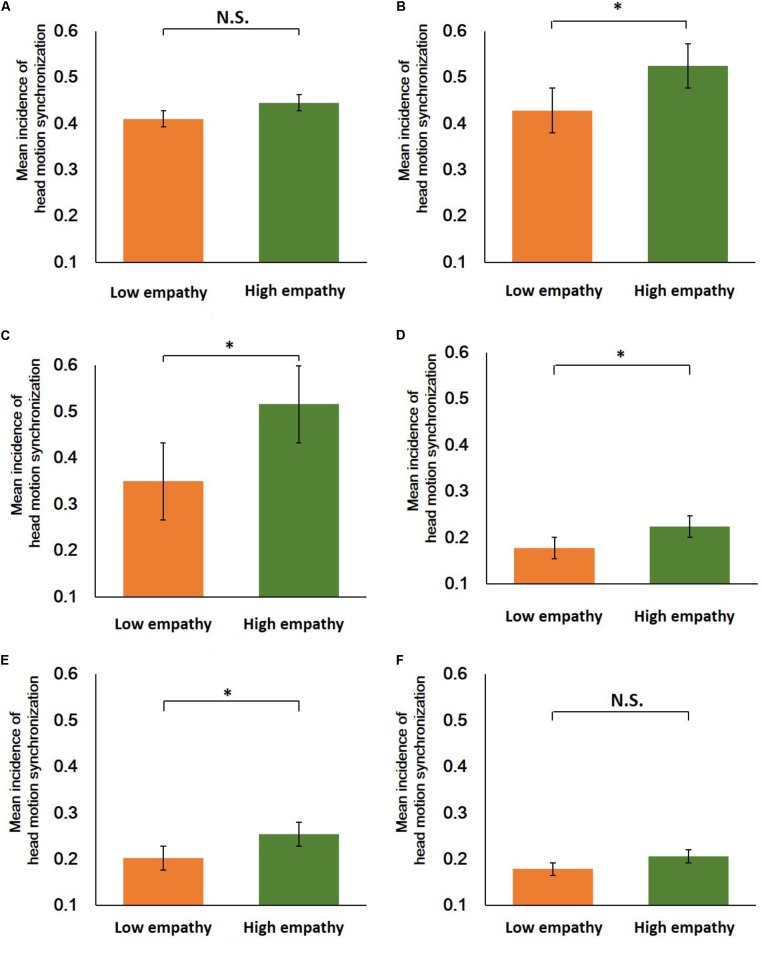
Wilcoxon’s signed rank test for low and high empathy conditions for each area in **Figure 6**. **(A)** Area a (*n* = 23, *Z* = –1.73, N.S.). **(B)** Area b (*n* = 23, *Z* = –3.32, ^∗^*p* < 0.01). **(C)** Area c (*n* = 23, *Z* = –3.38, ^∗^*p* < 0.01). **(D)** Area d (*n* = 23, *Z* = −3.32, ^∗^*p* < 0.01). **(E)** Area e (*n* = 23, *Z* = –3.15, ^∗^*p* < 0.01). **(F)** Area f (*n* = 23, *Z* = –2.47, N.S.).

After detection of head motion synchronization for each pair, the length of utterance of each pair was adjusted. Then, the mean value of head motion synchronization for each phase and frequency for all pairs (**Figures 5**, **6**) was calculated. Finally, each condition was grouped into six groups of the same size, and a mean value significance test was conducted using Wilcoxon’s signed rank test for each group (**Figure 7**).

## Results

### Head Motion Synchronization Analysis

**Figure 5** shows the results of a head motion synchronization analysis, where the horizontal axis represents frequency and the vertical axis represents the synchronization phase difference between the speaker and listener. On the vertical axis, positive values indicate that the listener’s head moved an instant before the speaker’s, while negative values mean the converse. In this analysis, we performed a STFT analysis to obtain the power spectrum of the acceleration norm of participants’ head motions from time-series data on head motion acceleration. We then calculated the Spearman’s rank correlation coefficient to identify head motion synchronization with a binary label, where “1” indicates synchronization” and “0” means no synchronization. Finally, we calculated the mean value incidences of head motion synchronization of the 23 pairs from the time-series data. In the figures, the incidence of head motion synchronization is illustrated by a continuous spectrum of colors from red to blue. **Figure 5A** shows the incidence of head motion synchronization during the low empathy condition. Here, the incidence of head motion synchronization had large values in the region from ∼1.0 to ∼3.0 Hz and from 0.0 s to about −0.50 s in phase difference. **Figure 5B** shows the incidence of head motion synchronization during the high empathy condition. Here, the incidence of head motion synchronization had large values in the region from ∼1.5 to ∼5.0 Hz in frequency and from ∼0.2 s to about −0.50 s in phase difference.

**Figure 6** shows differences in the incidence of head motion synchronization between the low and high empathy conditions. Areas with relatively large positive differences in the incidence of head motion synchronization in the high-frequency region are evident. Next, we divided this figure into six representative areas to confirm the distribution tendency of synchronization during the high and low empathy conditions. From a physical viewpoint, frequency represents the speed of head motion, which is commonly divided into low, middle, and high areas. On the other hand, phase difference represents the temporal order of participants’ head motions and is therefore reasonably divided into positive and negative values. This grouping helps us interpret the differences in the phase and frequency synchronization relationships in head motion corresponding to different listeners’ empathic states from the viewpoint of perception in face-to-face communication.

**Figure 7** shows the Wilcoxon’s signed rank test for each area. A *p*-value < 0.01 was considered statistically significant (^∗^*p* < 0.01). This result indicates that in the negative phase relationship areas, speaker-led head motion synchronization with medium (area b) and high (area c) frequencies was statistically more common during high empathy (area b: *n* = 23, *Z* = −3.32, ^∗^*p* < 0.01; area c: *n* = 23, *Z* = −3.38, ^∗^*p* < 0.01). In contrast, in the positive phase relationship areas, listener-led head motion synchronization at every frequency was statistically more frequent in the high empathy part (area d: *n* = 23, *Z* = −3.32, ^∗^*p* < 0.01; area e: *n* = 23, *Z* = −3.15, ^∗^*p* < 0.01).

## Discussion

To investigate the hypothesis that the phase and frequency relationships of body motion synchronization change according to the degree of empathy during face-to-face communication, we conducted a lecture task experiment using a controlled script divided into high and low empathy sections. During the randomly assigned speaker’s lecture to a listener, we measured the acceleration of both speaker and listener head motions using an accelerometer, and analyzed their synchronization from the time-series data on acceleration norms. The statistical analyses in **Figure 7** show that head motion synchronization between the speaker and the listener occurred more often during the high empathy portion of the lecture compared with the low empathy portion, in all areas except a and f. In other words, in the positive phase difference region where the speaker’s phase leads, there was a significant difference in the incidence of head motion synchronization within a medium frequency area (area b) and a high frequency area (area c). However, in the negative phase difference region where the listener’s phase leads, there were significant differences in incidence within the low frequency area (area d) and middle frequency area (area e). In addition, **Figure 5B** shows that during high empathy, speakers’ head motions started earlier compared with listeners,’ although there were also cases in which speakers started about 0.1–0.2 s later than the listeners.

Based on these results, we focus on the relationship between the phase and frequency relationship of head motion synchronization and the degree of empathy. A listener’s head motions are known to indicate their comprehension of communication content and to prompt the speaker to make the next utterance, thus encouraging smooth communication (Eibl-Eibesfeldt, 1972; Morris, 1977). In contrast, a speaker’s head motions are often seen in conjunction with catching the listener’s eye at the end of an utterance, to confirm that the listener has heard the utterance and understood its content (Hadar et al., 1983). Furthermore, head motions are a conversational behavior used to show politeness based on a communication strategy (Ohashi, 2013). In our experiment, the high empathy condition consisted of more concrete, everyday, easily understood sentences compared with the low empathy condition. Therefore, the high empathy section induced head motion synchronization between the speaker and listener to facilitate smooth conversation. This is consistent with the report that conversation smoothness was experienced during synchronized conversation (Chartrand and Bargh, 1999).

Regarding the relationship between the head motion synchronization phase difference and degree of empathy, **Figures 5B**, **7** show that compared with low empathy, during high empathy the speaker-led synchronization occurred more frequently within a middle frequency area (area b: 2.5 to 3.5 Hz) and a high frequency area (area c: 4.0 to 5.0 Hz). This suggests that when a listener is in a highly empathic state, high frequency head motion might occur because it signals empathy with the story being read aloud by the speaker. Thus, if the speaker perceives the listener’s high frequency head motions, they might perceive the listener’s interest in addition to their understanding of the content.

Furthermore, we found that listener-led synchronization occurred more frequently during the high empathy part of the lecture compared with the low empathy part (see areas d and f **Figures 6**, **7**). This suggests the possibility that high empathy content may enhance listeners’ predictive head motions, which send a positive signal about comprehension and interest, and make the speaker comfortable. Probably, the speaker would feel a comfortable gap between utterances because it was possible to receive a positive signal that the listener understood without a long interval for confirmation. Indeed, it has been shown that during telephone dialogs, participants’ predictive movements were important to their temporal comfort (Campbell, 2007). In other words, in a state in which the listener’s degree of empathy was high, it is conceivable that the listener predicted the timing of events such as sentence breaks, when the speaker generated the head motion for confirmation and the listener made the head motion according to this timing. As an example of this type of timing of non-verbal motion, the occurrence of blinking moves toward synchronizing with the end of an utterance (Nakano and Kitazawa, 2010). These reports have a similar tendency as listeners’ predictive head motions gave speakers a positive impression in unidirectional communication, such as the lecture task in this study.

In this study, we investigated the relationship between head motion synchronization and degree of empathy, and inferred that the degree of empathy might be reflected in the phase and frequency relationship of head motion synchronization. However, there are some limitations to our study. (1) Our definition of empathy. Although we used this term in relation to the lecture material, empathy has many definitions that encompass a wide range of emotional states, including empathy for another person. Therefore, in future work, it is necessary to establish a new experimental setting to examine the listener’s degree of empathy with the speaker. (2) Dependence on the content of utterances. Similar experiments should be conducted with different lecture material to determine whether similar results are found. (3) Directionality of speech and restrictions of a script. In daily life, dialog is typically bidirectional and usually without a script; hence, descriptive understanding such as used in this study becomes more challenging. (4) Culture dependence of non-verbal behavior. Since non-verbal behavior is highly culture-dependent (Eibl-Eibesfeldt, 1972), it is desirable to investigate whether these results are universal across different cultures. (5) Selection of the physical movement for quantification. Here, we analyzed frequency and phase difference based on head motion acceleration. However, considering that synchronization and empathy are types of cognitive states that emerge through complex human communication, other observable behaviors should be considered. Overcoming these limitations would provide a deeper understanding of the specifics of non-verbal behavior in human communication and lead to pursuing more comfortable human–human interactions.

## Author Contributions

TY designed the experiment, collected and analyzed head motion and empathy data, and wrote the paper. EO and YI designed the experiment and provided the linguistic structure data analysis. K-IO provided conceptual advice regarding the experiments and results. YM supervised the study and experimental design. All authors discussed the results and commented on the manuscript.

## Conflict of Interest Statement

The authors declare that the research was conducted in the absence of any commercial or financial relationships that could be construed as a potential conflict of interest.

## Supplementary Material

APPENDIX 1 Experiment lecture script, adapted from Wikipedia’s (2013) Japanese article “The meaning of life.”

## References

[B1] Bavelas J. B. Black A. Lemery C. R. Mullett J. (1986). I show how you feel: motor mimicry as a communicative act. *J. Pers. Soc. Psychol.* 50 322–329. 10.1037/0022-3514.50.2.322

[B2] Bernieri F. J. (1988). Coordinated movement and rapport in teacher–student interactions. *J. Nonverbal Behav.* 12 120–138. 10.1007/BF00986930

[B3] Bernieri F. J. Reznick S. Rosenthal R. (1988). Synchrony, pseudosynchrony, and dissynchrony: measuring the entrainment process in mother-infant interactions. *J. Pers. Soc. Psychol.* 54 243–253. 10.1037/0022-3514.54.2.243

[B4] Campbell N. (2007). “Approaches to conversational speech rhythm: speech activity in two-person telephone dialogues,” in *Proceedings of the International Congress Phonetic Science*, Saarbrücken, 343–348.

[B5] Chartrand T. L. Bargh J. A. (1999). The chameleon effect: the perception-behavior link and social interaction. *J. Pers. Soc. Psychol.* 76 893–910. 10.1037/0022-3514.76.6.893 10402679

[B6] de Waal F. B. (2007). “The Russian doll model of empathy and imitation,” in *On Being Moved: from Mirror Neurons to Empathy*, ed. Braten S. (Amsterdam: John Benjamins Publishing Company), 49–69. 10.1075/aicr.68.06waa

[B7] Duffy K. A. Chartrand T. L. (2015). Mimicry: causes and consequences. *Curr. Opin. Behav. Sci.* 3 112–116. 10.1016/j.cobeha.2015.03.002

[B8] Eibl-Eibesfeldt I. (1972). “Similarities and differences between cultures in expressive movements,” in *Non-verbal Communication*, ed. Hinde R. A. (Princeton: Cambridge University Press), 297–314.

[B9] Fraisse P. (1982). “Rhythm and tempo,” in *Psychology of Music*, ed. Deutsch D. (London: Academic Press), 149–180. 10.1016/B978-0-12-213562-0.50010-3

[B10] Hadar U. Steiner T. J. Grant E. C. Clifford Rose F. (1983). Kinematics of head movements accompanying speech during conversation. *Hum. Mov. Sci.* 2 35–46. 10.1016/0167-9457(83)90004-0

[B11] Hall N. R. Millings A. Boucas S. B. (2012). Adult attachment orientation and implicit behavioral mimicry. *J. Nonverbal Behav.* 36 235–247. 10.1007/s10919-012-0136-7

[B12] Kato T. Takahashi E. Sawada K. Kobayashi N. Watanabe T. Ishi T. (1983). A computer analysis of infant movements synchronized with adult speech. *Pediatr. Res.* 17 625–628. 10.1203/00006450-198308000-00004 6889004

[B13] Koehne S. Hatri A. Cacioppo J. T. Dziobek I. (2016). Perceived interpersonal synchrony increases empathy: Insights from autism spectrum disorder. *Cognition* 146 8–15. 10.1016/j.cognition.2015.09.007 26398860

[B14] Komori M. Nagaoka C. (2008). Body movement synchrony in psychotherapeutic counseling: a study using the video-based quantification method. *IEICE Trans. Inf. Syst.* 6 1634–1640. 10.1093/ietisy/e91-d.6.1634

[B15] Komori M. Nagaoka C. (2011). The relationship between body movements of clients and counsellors in psychotherapeutic counselling: a study using the video-based quantification method. *Jpn J Cogn. Psychol.* 8 1–9.

[B16] Koole S. L. Tschacher W. (2016). Synchrony in psychotherapy: A review and an integrative framework for the therapeutic alliance. *Front. Psychol.* 7:862. 10.3389/fpsyg.2016.00862 27378968 PMC4907088

[B17] Koss T. Rosenthal R. (1997). Interactional synchrony, positivity, and patient satisfaction in the physician-patient relationship. *Med. Care* 35 1158–1163. 10.1097/00005650-199711000-00007 9366894

[B18] Lafrance M. Broadbent M. (1988). Group rapport: posture sharing as a nonverbal indicator. *Group Organ. Stud.* 1 328–333. 10.1177/105960117600100307

[B19] Lakens D. Stel M. (2011). If they move in sync, they must feel in sync: movement synchronization leads to attributions of rapport and entitativity. *Soc. Cogn.* 29 1–14. 10.1521/soco.2011.29.1.1

[B20] Lakin J. L. (2006). “Automatic cognitive progress and nonverbal communication,” in *The Sage Handbook of Nonverbal Communication*, eds Manusov V. L. Patterson M. L. (Thousand Oaks, CA: Sage Publications, Inc), 59–77. 10.4135/9781412976152.n4

[B21] Mehrabian A. Williams M. (1969). Nonverbal concomitants of perceived and intended persuasiveness. *J. Pers. Soc. Psychol.* 13 37–58. 10.1037/h0027993 5352374

[B22] Meltzoff A. N. Moore M. K. (1983). Newborn infants imitate adult facial gestures. *Child Dev.* 54 702–709. 10.2307/1130058 6851717

[B23] Miller N. Maruyama G. Beaber-Rex J. Valone K. (1976). Speed of Speech and Persuasion. *J. Pers. Soc. Psychol* 34 615–624. 10.1177/0146167218787805 30084307

[B24] Morris D. (1977). *Manwatching: A Field Guide to Human Behavior.* New York, NY: H. N. Abrams, Inc.

[B25] Nakano T. Kitazawa S. (2010). Eyeblink entrainment at breakpoints of speech. *Exp. Brain. Res.* 4 577–581. 10.1007/s00221-010-2387-z 20700731

[B26] Niedenthal P. M. (2007). Embodying emotion. *Science* 316 1002–1005. 10.1126/science.1136930 17510358

[B27] Ohashi J. (2013). *Thanking and Politeness in Japanese.* London: Palgrave Macmillan 10.1057/9781137009876

[B28] Ramseyer F. Tschacher W. (2006). Synchrony: a core concept for a constructivist approach to psychotherapy. *Constr. Hum. Sci.* 11 150–171.

[B29] Ramseyer F. Tschacher W. (2014). Nonverbal synchrony of head-and-body movement in psychotherapy: different signals have different associations with outcome. *Front. Psychol.* 5:979. 10.3389/fpsyg.2014.00979 25249994 PMC4155778

[B30] Richmond V. P. McCroskey J. C. Payne S. K. (2008). *Nonverbal Behavior in Interpersonal Relations.* Boston: Pearson/Allyn and Bacon.

[B31] Stephens G. J. Silbert L. J. Hasson U. (2010). Speaker–listener neural coupling underlies successful communication. *Proc. Natl. Acad. Sci. U.S.A.* 107 14425–14430. 10.1073/pnas.1008662107 20660768 PMC2922522

[B32] Thepsoonthorn C. Yokozuka T. Miura S. Ogawa K. Miyake Y. (2016). Prior knowledge facilitates mutual gaze convergence and head motion synchronization in face-to-face communication. *Sci. Rep.* 6:38261. 10.1038/srep38261 27910902 PMC5133623

[B33] Tickle-Degnen L. Rosenthal R. (1990). The nature of rapport and its nonverbal correlates. *Psychol. Inq.* 1 285–293. 10.1207/s15327965pli0104_1

[B34] Wikipedia’s (2013). Meaning of life. Available at: https://ja.wikipedia.org/wiki/%E4%BA%BA%E7%94%9F%E3%81%AE%E6%84%8F%E7%BE%A9

[B35] Yun K. Watanabe K. Shimojo S. (2012). Interpersonal body and neural synchronization as a marker of implicit social interaction. *Sci. Rep.* 2:959. 10.1038/srep00959 23233878 PMC3518815

