# Supplementary material for: The Relationship Between Head Motion Synchronization and Empathy in Unidirectional Face-to-Face Communication
Source: Front Psychol. 2018 Sep 25;9:1622. doi: 10.3389/fpsyg.2018.01622 (PMC6167983; doi:10.3389/fpsyg.2018.01622)
Supplement: APPENDIX 1 — Experiment lecture script, adapted from Wikipedia’s (2013) Japanese article “The meaning of life.” [file Data_Sheet_1.pdf]

Appendix 1. Lecture Script

I will explain the "meaning of life" that has been academically considered by our predecessors.

High Empathy Part

First of all, the meaning of life concerns the question of whether there is a purpose or a meaning to life. If you say there is a meaning to life, then what is it?

In real society, this question becomes a more serious problem in economically rich countries.

In economically and materially rich countries, the number of people who are lost to a terrible "emptiness of mind" is on the increase.

As a reason for this, psychologists argue that humans must satisfy all of their basic needs, such as clothing, food, and living, before they begin to feel deprived of higher desires such as a "desire for self-realization."

Because "a rich society" is one where it is easier to satisfy basic needs, it is easier for high-level desires to manifest and for people to suffer from not satisfying these high-level desires.

Also, it is said that there are three periods of life when such thoughts capture the hearts of people: puberty, middle age, and old age.

Many who have gone through puberty have answers at that stage, but such questions may also occupy the mind during middle age or old age.

When you were young, you thought, "I will be happy if I acquire property, position, family, etc." If you tried hard and obtained all of these, you realized that you were not

completely happy. This means that when you feel that “something important” is missing in life, you feel aware of “the vanity of life.”

At this stage, you directly face the question, “How can I live in the future to make life more meaningful?”

Sociologists insist that this question concerns determining one’s own values or attitudes toward life; as such, academics and science do not provide answers to this question.

On the other hand, we can consider the following ideas.

Low Empathy Part

In the field of philosophy, there have been many discussions about this question.
For example, a logical positivist asks “What is the meaning of life?” and “Is there a meaning to ask about?”

The idea here is “If this objective value does not exist, is life meaningless?”.

Ludwig Wittgenstein and logical positivists argued that “if expressed by language, that question is meaningless.”

Jeremy Bentham, the founder of Utilitarianism, argued that being under the two rulers of pleasure and unpleasantness is human nature. He developed the theory of dominance of utilitarianism from moral insights and “good is whatever is the greatest happiness of the greatest number.”

Bentham defined the meaning of life as “the principle of maximum happiness.”

On the other hand, the idea of nihilism argues that life has no meaning.

Nihilism is a process of “making the highest value worthless.” Friedrich Nietzsche said that the characteristic of nihilism is to empty the world and human existence of meaning, existence, purpose, intellectual truth, and essential value.

Martin Heidegger said that nihilism is a movement in which “existence” is forgotten, transforming existence into value, reducing the existence to economic exchange.

Thus, over time, the question about the meaning of life has generated many philosophical arguments and ideas.

This completes the lecture.
